# Differences in substance use by sexual orientation and gender among Jewish young adults in Israel

**DOI:** 10.1186/s13584-020-00410-4

**Published:** 2020-10-07

**Authors:** Hagit Bonny-Noach, Mally Shechory-Bitton

**Affiliations:** The Department of Criminology, Ariel University, Ariel, Israel

**Keywords:** Substance use, Sexual orientation, Gender differences, Young adults, Homosexuals, Peers, Israel

## Abstract

**Background:**

This study focuses on sexual orientation and gender-based differences among Israeli young adult substance use behaviors. In addition, it evaluates young adult perception of substance use and acceptance of substances use by close friends.

**Methods:**

We conducted a cross-sectional study. A self-reported anonymous questionnaire was distributed to a convenience sample of 496 young-adults (age: M = 23.14, SD = 2.48), which included 126 heterosexual males, 128 heterosexual females, 131 gay men, and 111 lesbians.

**Results:**

This study revealed significant sexual orientation and gender differences in all outcomes examined. Significant substance usage differences were found for same-sex orientation as 52% reported cannabis use and 24% reported using other illegal substances during the past 12 months compared to 34 and 6% (respectively) among heterosexuals. Significant gender differences were found, as male participants reported 50% cannabis use and 19% reported other illegal substance use in the past 12 months compared to 35 and 11% (respectively) among females.

Additionally, compared with heterosexuals, gay men and lesbians perceived/assessed significantly higher substance usage rates among their close friends and higher levels of substance use acceptance by close friends. Regression models indicated the important role of respondent perceived and acceptance of substance use among close friends.

Binge drinking, cannabis use, and other illegal substance use were positively associated with participants’ perceived substance use and substance use acceptance level by close friends, after controlling for gender, sexual orientation, age, and level of education.

**Conclusions:**

Close friends and community norms can play an important role in shaping substance usage among young adults, especially among gay men and lesbians. The results of the current study highlight the need for developing prevention and harm reduction drug policies for Israeli young adults, especially for gay men and lesbians. Interventions should also focus on young adult peers and community norms related to substance use by professionals in educational, policy-making, and therapeutic contexts.

## Introduction

Substance use is commonly reported in same-sex relationships, with higher usage rates in the young adult lesbian and gay men (LG) community [1, 2] than among heterosexuals [3–5]. Focusing on gender differences shows a clear pattern of higher substance use among males [6–8]. However, the results for gay men and lesbians were more ambiguous, depending on substance type [1, 9, 10].

Higher substance use in LG communities is often linked to minority-specific stressors, including victimization, discrimination, stigma, and rejection [11–13]. Other studies report a shared set of LG values, of which ‘normalization’ of substance use is one [14, 15]. Substance use is often considered normative behavior by LG youths in gay men identified venues (e.g., pubs, dance clubs, etc.). It serves as a way to affirm a sense of belonging in the LG community [16, 17]. In addition, higher rates of heavy alcohol and binge drinking characterize gay men’s community activities [11]. Patterns of sexualized substance use are mostly experiences of men who have sex with men, which has been labeled ‘chemsex’ and received increasing research attention mostly in Europe [18, 19] and in recent years also in Israel [20, 21].

It is well established that substance use is affected by social context. Some substances are more used in social settings with peers, and this phenomenon has been studied in heterosexual adolescent populations. This research shows that peers may function as important socialization agents that directly affect adolescent norms regarding risk behaviors, including substance use [22, 23]. Adolescents are more likely to adopt substance use norms and behaviors from socialization agents with whom they share strong relational bonds [24]. They tend to befriend as well as choose to affiliate with peers whose attitudes and behaviors resemble their own on alcohol use [25–27]. The same socialization pattern applies to smoking [28] and drug use [29]. Individuals also tend to overestimate the substance use of their peers, resulting in their own higher levels of substance use [30, 31]. However, most research on peer influence on substance use has been conducted on heterosexual adolescents. There is thus a lack of knowledge on the subject of peer influence on substance use in young adults and, in particular, the LG community in Israel.

In Israel, rates of substance use and binge drinking are highest among young adults, with the average age of first use of illegal drugs almost 22 years old [32]. At this age, many young adults finish their mandatory army service and choose to travel abroad on backpacking trips, where they are exposed to high levels of drug use [33–35]. A National Epidemiological Survey published in 2017 [32] reported that for young adults aged 18–34, over 40% use cannabis and more than 3% have used any other illegal substance in the past year. However, this survey does not ask about sexual orientation, and there is thus a lack of data on the LG population in Israel.

Due to the unique characteristics of Israeli society, it is noteworthy to examine substance use among Jewish LG young adults in Israel. Even though Israel boasts a relatively open LG community, there are many who still conceal their sexual identity [36]. Israel enjoys various nondiscriminatory laws and regulations, and, in the past decade, services to meet the needs of LG adolescents and young adults [37]. Nevertheless, LG are still exposed to homophobic and negative attitudes, and this extends to the way they express their gender identity [38]. Young people aged 18 (boys and girls) are required to perform mandatory military service. For LG individuals, military service is a common stressor mainly because the army idealizes hegemonic masculinity [39]. However, this also likely allows LG youth to become more independent adults [40].

Most research on LG substance use is conducted in Western countries [41–43]. However, Israel represents a unique case study, and, as far as we know, only a small number of studies also included substances use among the LG community [20, 21, 44]. This body of research focused mostly on health risk behaviors, including substance use patterns. One of these studies looked at the variety of risk behaviors, including substance use among Israeli LG young adults (aged 16–23). Their results show that LG participants engaged in more physical risk behaviors (e.g., smoking, substance use, and risky sexual behaviors) than their heterosexual counterparts [44]. Other studies reveal that recreational drug use, mostly during sex, is associated as a risk factor with increases in HIV diagnoses among Israeli gay men [21].

The goal of the current study is to evaluate substance use in Israeli young adults based on sexual orientation and gender differences. We also assess perceptions/ assessments of substance use by, and its level of acceptance among, close friends. This research has the following objectives: First, it assesses prevalence of and differences in substance use among Israeli LG and heterosexual young adults, as well as gender effects. Second, it investigates perceived substance use by and acceptance among their close friends. This research also examines predictors of cannabis use and problematic forms of consumption such as binge-drinking and illegal substance use among Israeli LG and heterosexual young adults, focusing on differences based on sexual orientation and gender. Following the literature review, we hypothesize that sexual orientation, gender, younger age, and perceived substance use and acceptance among close friends will predict consumption habits of binge drinking, cannabis use, and other illegal substance use.

## Methods

### Participants

The target convenience sample size included 496 young adults, with 254 participants who identified as heterosexual (126 males and 128 females), 131 who identified as gay men, and 111 who identified as lesbians.

### Instruments

The following questionnaires were used in this study:

- ***A self*-*report questionnaire on psychoactive substance use*** – the questionnaire consisted of questions on own substance use , *perceived substance use by close friends* and *acceptance of substance use among close friends*. These questions were adapted from the 2009 National Epidemiological Survey carried out by the Israel Anti-Drug Authority [45]. The following variables were used to assess reports of current *own-use*, all coded as yes or no: (1) cigarettes, (2) beer, (3) hard alcohol/spirit, and (4) binge drinking (heavy episodic use of alcohol consisting of consuming five or more alcoholic beverages in the span of a few hours). Each was defined as 1 (using) and 0 (no use).

Two questions examined the respondent and friend *cannabis use*: "Have you/your friends used cannabis?" Response options included (1) never used, (2) past 30 days, and (3) past 12 months. Each was defined as 1 (yes) and 0 (no).

For *own-use* and *perceived 'Other substance use' by close friends* over the past 12-months, perceived variables were used, all coded as yes or no: (1) 'Hagigat' (i.e., the street name for increasingly common NPS-amphetamine-type stimulants), (2) New psychoactive substances (Designer synthetic Cannabinoid types of NPS also called ‘herbal highs’ or 'sam pizuziot' in Israel ), (3) MDMA(Methylenedioxymethamphetamine), (4) LSD, (5) nitrite inhalants ("Poppers"), (6) Cocaine, (7) Ketamine, (8) Mushroom, (9) Cactus, and (10) Heroin. Participants and their friends were regarded as using drugs in the past year based on a positive response for any of these substances.

One question examined respondent *acceptance of substance use among close friends*: "If your friends thought you were using illicit drugs regularly, how would they react?" Response options included: (1) They would accept it; (2) They would not care; (3) They would oppose it, but continue to be my friends; and (4) They would oppose it, and cease to be my friends. This variable was defined dichotomously as 1 (would accept or not care) and 0 (would oppose).

- ***A demographic questionnaire including questions on sexual orientation*** – *A socio-demographic questionnaire* consisting of demographic details: gender, age, family status, education, employment status, and questions on sexual orientation focused on assessment of sexual identity by asking "How would you define your sexual orientation?" Response options included (1) heterosexual, (2) homosexual, and (3) bisexual.

### Procedure

This study was approved by the ethical standards of the Institutional Review Board (IRB) of the University. We conducted a cross-sectional study. Four young-adult research assistants were recruited: two lesbian, one gay male, and one heterosexual female. They distributed the questionnaire among young adults in paper format. A snowballing method was also used, as respondents were asked to relay the questionnaire to friends based on age requirement. In addition, owing to the difficulty of obtaining a representative Israeli LG sample [44] the research assistants distributed the questionnaire to gays and lesbians in settings where LGs gather (e.g., Meir Garden in Tel Aviv, LGBT groups on university and college campuses). Additionally, due to the difficulty of obtaining a gay male sample, a gay male research assistant distributed the questionnaire via e-mail to 49 gay men. No differences were found in the comparison of the two groups based on manual or e-mail collection. Participants were asked to complete, on a voluntary and anonymous basis, the questionnaire in Hebrew. They were told that they were not required to complete the questionnaire if for whatever reason they were not interested in doing so. Following collection of the 506 questionnaires representing the entire sample, 10 were not filled out and therefore rejected.

### Data analysis

Data were analyzed with SPSS ver. 25. Gender differences and differences by sexual orientation for the dichotomous variables were analyzed with Z tests for independent proportions. Differences by gender and sexual orientation (4 groups) were analyzed with Kruskal-Wallis chi-square (χ^2^) tests, followed by Mann-Whitney U tests for significant chi-square (χ^2^) results. The 4 group difference for age was analyzed with a one-way analysis of variance. Multiple logistic regression models were analyzed for binge drinking, cannabis consumption, and other substance use. Gender (1-male, 0-female), sexual orientation (1-heterosexual, 0-gay male/lesbian), age (standardized), and education level (1-academic education, 0-high school education), participant perception of cannabis use among close friends (1-yes, 0-no), participant perception of other substance use among close friends (1-yes, 0-no), and participant acceptance of other substance use by close friends (1-accept, 0-oppose) were entered into the multiple logistic regression. The interaction of gender with the perceived substance use by close friends was examined in each of these regression models. Simple slopes analysis was used to interpret it when found significant (data not shown in table).

Sample size was calculated according to Peduzzi et al.’s (1996) equation for logistic regression models [46] (see Appendix).

## Results

Heterosexual females were the youngest in age. However, all the participants were in their early twenties and half of them were male. They mostly single and mostly had a high school education and were employed. Significant differences were found by gender and sexual orientation as a result of the large sample, yet these do not represent essential disparities. Significant sexual orientation differences were found according to family status, yet most respondents were single. In addition, most respondents have a high school education and were employed, with no significant sexual orientation difference. Results of demographic comparisons are reported in Table 1.

**Table 1:** Socio-demographic characteristic sub-groups by sexual orientation (*N* = 496)

	Total *N* = 496	Heterosexual male	Heterosexual female	Gay men	Lesbian	
	M (SD)	M (SD)	M (SD)	M (SD)	M (SD)	
**Age**	23.14 (2.48)	23.55 (2.19)	22.18 (1.90)	23.56 (2.97)	23.28 (2.47)	F(3, 492) = 9.42, *p* < .001, η2 = .054.
**Gender**						
**Male**	257 (52%)	126 (25%)	–	131 (26%)	–	
**Female**	239 (48%)	–	128 (26%)	–	111 (22%)	
**Family status**						
**Single**	383 (78%)	104 (83%)	112 (88%)	100 (77%)	67 (61%)	χ2(3) = 27.16, *p* < .001
**Not single**	110 (22%)	21 (17%)	16 (13%)	30 (23%)	43 (39%)	
**Education**						
**High school**	329 (70%)	84 (71%)	98 (79%)	77 (62%)	70 (67%)	χ2(3) = 11.33, *p* = .010
**Academic**	143 (30%)	34 (29%)	26 (21%)	48 (38%)	35 (38%)	
**Employment status**						
**Employed**	338 (68%)	77 (61%)	85 (66%)	98 (75%)	78 (71%)	χ2(3) = 6.12, *p* = .106
**Unemployed**	157 (32%)	49 (39%)	43 (34%)	33 (25%)	32 (29%)	

### Prevalence of substance use by sexual orientation and gender

As can be seen from Table 2, significant differences in substance use were found by sexual orientation. Usage rates were significantly higher for gay men and lesbian women than heterosexuals. These differences were smallest for consumption of beer and greatest for illegal drugs (with the exception of cannabis). In addition, significant differences were found by gender. Men had higher rates of substance use than women. These gender differences were smallest for beer drinking and greatest for binge drinking. Significant differences in substance use were also found within sexual orientation subgroups. Heterosexual females had the lowest rates of substance use. Almost no significant differences were found between gay men and lesbian women (only binge drinking over the past month had higher rates for gay men than lesbian women).

**Table 2:** Substance use by sexual orientation and gender (*N* = 496)

	Sexual orientation			Gender			Sexual orientation and gender				
	Hetero-sexual N (%)	Homo-sexual N (%)	Z	Male N (%)	Female N (%)	Z	1.Hetero-sexual male	2.Hetero-sexual female	3.Gay men	4.Lesbian	χ^**2**^(3)
**Cigarette smoking**	112 (44)	147 (62)	3.83***	151 (59)	108 (46)	2.93**	78 (62)	34 (27)	73 (56)	74 (69)	50.34*** 1,3,4 > 2
**Beer**	202 (81)	203 (84)	0.99	221 (86)	184 (78)	2.32*	112 (89)	90 (72)	109 (83)	94 (85)	13.24*** 1,3,4 > 2
**Hard alcohol/ Spirits**	180 (73)	204 (85)	3.17**	214 (85)	170 (72)	3.30***	103 (84)	77 (62)	111 (85)	93 (84)	27.36*** 1,3,4 > 2
**Binge-drinking- past 30 days**	77 (30)	107 (44)	3.17**	123 (48)	61 (26)	5.11***	57 (45)	20 (16)	66 (50)	41 (37)	38.18*** 1,3,4 > 2 3 > 4
**Cannabis - ever used**	119 (47)	155 (65)	4.04***	158 (62)	116 (49)	2.86**	71 (57)	48 (38)	87 (67)	68 (63)	26.04*** 1,3,4 > 2 3 > 1
**Cannabis - past 12 months**	85 (34)	125 (52)	4.12***	127 (50)	83 (35)	3.31***	53 (43)	32 (25)	74 (57)	51 (47)	27.31*** 1,3,4 > 2 3 > 1
**Cannabis - past 30 days**	57 (28)	92 (38)	3.73***	91 (36)	58 (24)	2.76**	36 (29)	21 (17)	55 (42)	37 (33)	20.81*** 1,3,4 > 2 3 > 1
**Any illegal drug (with exception of cannabis)past 12 months**	15 (6)	59 (24)	5.77***	48 (19)	26 (11)	2.43*	12 (10)	3 (2)	36 (28)	23 (21)	37.99*** 3,4 > 1,2

*p < .05, **p < .01, ***p < .001

### Participant perceived substance use by, and acceptance of substance use, among close friends, based on sexual orientation and gender

As can be seen from Table 3, gay men and lesbian women reported higher rates of substance use by close friends than heterosexual participants. Similarly, males reported higher rates of substance use by close friends than females (regardless of sexual orientation). This tendency is stronger for the use of all illegal drugs than cannabis. Acceptance of substance use by close friends was higher for gay men, lesbian women, and heterosexual men than heterosexual women.

**Table 3:** Perception of substance use and acceptance of substance use by close friends based on sexual orientation and gender

	Sexual orientation			Gender			Sexual orientation and gender				
	Hetero-sexual N (%)	Homo-sexual N (%)	Z	Male N (%)	Female N (%)	Z	1.Hetero-sexual male	2.Hetero-sexual female	3.Gay men	4.Lesbian	χ^**2**^(3)
**Perception of Cannabis use by close friends - past 12 months (*N*** **= 496)**	178 (71)	203 (85)	3.63***	213 (84)	168 (71)	3.27**	98 (78)	80 (64)	115 (89)	88 (79)	23.33*** 3 > 1,4 > 2
**Perception of any illegal drug (with exception of cannabis) by close friends - past 12 months (*N*** **= 496)**	63 (25)	119 (49)	5.62***	110 (43)	72 (30)	2.92**	40 (32)	23 (18)	70 (53)	49 (44)	39.03*** 3,4 > 1 > 2
**Perception of acceptance of substance use by close friends (*N*** **= 489)**	83 (34)	125 (52)	4.11***	139 (55)	69 (29)	5.74***	64 (52)	19 (15)	75 (58)	50 (45)	55.14*** 1,3,4 > 2

**p < .01, ***p < .001

### Prediction of binge drinking, cannabis consumption, and illegal substance use

Logistic regressions were calculated to predict the dependent variables of the study: binge drinking (in the past 30 days), cannabis consumption (in the past 30 days), and other substance use (over the past 12 months). Independent variables included gender and sexual orientation (which compose the main sub-groups in this study), age and education (which were found different between the sub-groups), and perception of substance use and acceptance of substance use by close friends (which were defined as independent variables, being related to substance use) in this study.

The results reveal (Table 4) that the three models are significant. The likelihood of binge drinking in the past month was higher for males, participants with same gender sexual orientation, participants with a high school education, and participants who perceived their friends as both using cannabis and accepting of the use of other substances. The likelihood of cannabis use in the past 30 days was higher for participants who perceived their close friends as using cannabis and other substances, as well as accepting the use of other substances. The likelihood for other substance use over the past 12 months was higher for participants with same gender sexual orientation and participants who perceived their close friends as using other substances.

**Table 4:** Logistic regressions predicting binge drinking, cannabis use, and other substance use (except cannabis) with perception of cannabis use, substance use, and substance use acceptance by close friends (*N* = 463)

	Binge drinking		Cannabis use in the past month		Other substance use in the past 12 months	
	***B*** (***SE***)	***OR*** (95% ***CI***)	***B*** (***SE***)	***OR*** (95% ***CI***)	***B*** (***SE***)	***OR*** (95% ***CI***)
**Gender (male)**	0.98 (0.22)	2.66*** (1.72, 4.13)	0.18 (0.24)	1.20 (0.75, 1.90)	0.51 (0.31)	1.66 (0.90, 3.07)
**Sexual orientation (heterosexual)**	−0.50 (0.22)	0.61* (0.39, 0.94)	−0.39 (0.24)	0.68 (0.42, 1.08)	−1.33 (0.35)	0.26*** (0.13, 0.53)
**Age**	−0.09 (0.05)	0.91 (0.83, 1.00)	0.06 (0.05)	1.07 (0.97, 1.17)	−0.06 (0.06)	0.94 (0.84, 1.07)
**Education (academic)**	−0.58 (0.27)	0.56* (0.33, 0.94)	−0.08 (0.28)	0.92 (0.53, 1.59)	−0.26 (0.37)	0.77 (0.37, 1.60)
**Perception of cannabis use in the past 12 months by close friends**	1.42 (0.36)	4.14*** (2.06, 8.34)	2.22 (0.61)	9.20*** (2.76, 30.66)	0.78 (0.67)	2.18 (0.58, 8.15)
**Perception of substance use in the past 12 months by close friends**	0.33 (0.23)	1.39 (0.88, 2.19)	0.70 (0.24)	2.01** (1.26, 3.20)	2.13 (0.37)	8.42*** (4.06, 17.44)
**Perception of acceptance of substance use by close friends**	0.48 (0.23)	1.61* (1.02, 2.53)	0.95 (0.24)	2.58*** (1.60, 4.15)	0.19 (0.33)	1.21 (0.64, 2.30)
	χ^2^(7) = 95.27*** R^2^ = .254		χ^2^(7) = 107.71*** R^2^ = .296		χ^2^(7) = 101.67*** R^2^ = .346	

*p < 0.05, **p < 0.01, ***p < 0.001. R^2^ = Nagelkerke’s R^2^

#### Cannabis use in the past 30 days by gender and perceived other substance use by close friends

In order to assess the extent to which substance use by close friends is uniquely related with cannabis use among males versus females, the interaction was defined and entered in the last step of the regression. It was found significant: B = − 1.02, SE = 0.47, OR = OR = 2.78, *p* = .031, 95% CI = 1.10, 7.14. Perceived substance use by friends was related with higher cannabis use among females (B = 1.31, t = 3.52, *p* < .001), and not among males (B = 0.28, t = 0.95, *p* = .343).

## Discussion

In the current study, we focused on the association between sexual orientation and gender differences in Israeli young adults in order to assess binge drinking, cannabis use and other illegal substance use. We also evaluated their perceived substance use by close friends and the level of acceptance of substance use among close friends.

As expected, and consistent with previous studies, the current findings indicated that Israeli LG young adults reported higher rate of substance use than heterosexuals [1, 4, 5].

Minority-specific stressors, including victimization, discrimination, stigma, and rejection in LG communities is often linked to higher substance use [11–13]. In addition, in Israel, LG are still exposed to homophobic and negative attitudes, and this extends to the way they express their gender identity [38]. In the present study, we did not focus on the causes of substance use. But these can be assumed to be, at least in part, related to distress experienced by same-sex individuals in Israel. Despite the increase in awareness, Israeli society is still influenced by traditional perceptions of sexual orientation. These cause serious problems for same-sex individuals as there are considerable segments of Israeli society characterized by more traditional views of gender roles [36].

However, other studies also report a shared set of LG values, with ‘normalization’ of substance use one of them [14, 15] and substance use serving as a way to affirm a sense of belonging in the LG community [16, 17]. Thus, the findings in our regression models may suggest that the important role of perceived substance use and acceptance among close friends could strengthen the influence of social norms. Indeed, these were positively explained by perceived substance use by and acceptance of substance use among close friends controlling for sexual orientation or gender. Thus, belonging to a peer group and to a young adult community can play an important role in shaping substance usage among young adults in general and among gay men and lesbians in particular. Higher substance usage rates among the LG community may be seen as a way to affirm a sense of belonging and shared values and less as an extension of stressful pathways. These suggestive research insights should be explored in future studies that will focus on motivations for substance use among young adult LG.

One possible explanation based on gender differences that was found in this research and is consistent with previous studies [6, 7] may reside in cultural expectations and reflect gender roles. Men may experience greater motivation to drink alcohol than women as a means of demonstrating masculinity, facilitating aggression, exerting power, and taking risks [47]. In addition, there is an added patriarchal belief that female control over their social behavior (such as their sexuality) and ability to fulfill responsibilities is more precarious than males [48]. As Israeli society is based on traditional family values, such as high marriage rates, low divorce rates, and the importance of the family and motherhood [49, 50], these gender role divisions continue to remain relevant [51, 52]. Furthermore, Israeli traditionalism is buttressed by the religious establishment and dominance of the military in forming masculine identities [53], with the army playing a substantial role in sustaining hierarchical gender relations [54].

As noted, most research on peer influence on substance use has been conducted on heterosexual adolescents [30, 31]. This study suggests that young-adult peers may also function as important socialization agents determining substance use behaviors. As with adolescents, young adult likelihood of adopting substance use behaviors is based on socialization agents, especially those with whom strong relational bonds are shared [24].

Our findings also show the significant influence of perceived substance use by and acceptance of substance use among close friends on own-use. LG participants reported higher other substance acceptance among and use by close friends. Thus, close friends exert substantial influence in the LG community and may function as important socialization agents directly affecting substance use. This behavior among LG young-adults is mostly exhibited in any illegal “hard” drug use with the exception of cannabis, which is considered to be normative behavior among young adults in general [32].

Results from the regression models suggest that among LG, the higher rates of binge drinking, cannabis consumption, and other illegal substance use is positively explained by perceived substance use by and acceptance of substance use among close friends beyond sexual orientation. Higher rates of substance use are not only linked to minority-specific stressors as claimed in previous studies [11, 13], but represent a normative behavior, indicating willingness to engage in same-sex community activities [11, 16]. This research direction is recommended for further follow-up studies, to reveal peer influences based on substance use among young LG adults.

Our study is the first to demonstrate important findings based on the association between own-use and perceived substance use by and acceptance of substance use among close friends. Most research on the influence of close friends and peers tends to focus on heterosexual adolescents [22, 23].

A number of considerations limit the scope of our findings. First, there is potential lack of heterogeneity in the outcomes due to using snowball sampling. This was partly due to difficulty in the collection of data that required a sample consisting of an Israeli LG sample. Although this type of sampling is typical for studies of parents, owing to difficulties in recruiting research subjects [44], it may limit research generalizability to broader contexts. In addition, we focused on heterosexual versus gay and lesbian same-sexual preference. Other sexual dimensions exist such as bisexual, asexual, and transsexual. We also focused on sexual identity and not behavior. Using a more diverse sample as a comparison may contribute to the external validity of future results. Also, the study found a set of associations between a respondent’s own substance use and perceptions of friends’ substance use and attitudes and not a direction of causality. It is recommended to conduct a different and continuous research to examine direction of causality. And, finally, in this study, young adults were asked about perceived substance use by and acceptance of substance use among close friends, with a more recommended option being to inquire directly from close friends themselves.

## Conclusions

This study revealed significant sexual orientation and gender differences according to substances use. Even so, it is important to note that the LGBT community has embarked on a cooperative initiative with health and welfare representatives to develop harm reduction interventions, mostly in the Tel Aviv area [55]. There exists a strong need to develop coherent national drug prevention and harm reduction policies for Israeli young adults in general and for LG in particular. Additionally, the current findings suggested that close friends and community norms can play an important role in shaping substance usage among young adults in general and among gay men and lesbians in particular. Therefore, peer and community norm related substance use should be addressed by professionals in educational, policy-making, and therapeutic contexts. Additionally, the National Substances Use survey in Israel should routinely address sexual orientation.

## Data Availability

The authors have the data.

## Appendix

Sample size was calculated according to Peduzzi et al.’s (1996) equation for logistic regression models [45]. The formula is *N* = 10 k / p, where p = the rate of positive events in the population, and K = the number of independent variables in the logistic regression model. In a simulation study, Peduzzi et al.’s (1996) showed that this calculation is able to estimate a 5% difference in effect size, with an error rate of 5 and 80% power. According to this calculation, the minimum required sample for the regression of using other substances in the past 12 months (with the lowest rate of prevalence) is 470 participants. As the use of cannabis and binge drinking have a higher rate of prevalence, the required sample size for the analysis of their use in a logistic regression model is smaller.

## Abbreviations

LG: Lesbian and Gay men
LGBT: lesbian, gay, bisexual, and transgender
